# Supplementary material for: Oral Intake of L-Ornithine-L-Aspartate Is Associated with Distinct Microbiome and Metabolome Changes in Cirrhosis
Source: Nutrients. 2022 Feb 10;14(4):748. doi: 10.3390/nu14040748 (PMC8875633; doi:10.3390/nu14040748)
Supplement: Supplementary file 1 [file nutrients-14-00748-s001.zip › nutrients-1571629-supplementary.pdf]

Supplementary methods and results

Oral intake of L-ornithine-L-aspartate is associated with distinct microbiome and metabolome changes in cirrhosis

Angela Horvath[1,2], Julia Traub[3], Benard Aliwa[1], Benjamin Burgeois[4,5], Tobias Madl[4,5], Vanessa Stadlbauer[1,2]

1 Department of Gastroenterology and Hepatology, Medical University of Graz

2 Center of Biomarker Research in Medicine, CBmed GmbH Graz

3 Department of Clinical Medical Nutrition, University Hospital Graz

4 Gottfried Schatz Research Center for Cell Signaling, Metabolism and Aging Molecular Biology and Biochemistry, Medical University of Graz

5 BioTechMed-Graz, Graz, Austria

DNA extraction, 16s library preparation and sequencing

Total genomic DNA was extracted from stool samples using a MagnaPure LC DNA isolation kit protocol. 250µl of homogenized samples were transferred to Magna Lyser green bead tubes for mechanical lysis at 6500 rpm for 30 seconds two times in a MagNA Lyser Instrument (Roche, Mannheim Germany). This was followed by enzymatic lysis with 20µl lysozyme at 37°C for 30 minutes and 30µl Proteinase K for 1.5 hours at 65°C. Proteinase K was heat-inactivated at 95°C for 10 minutes. The remaining steps were performed according to Magna Pure kit protocol, and 250µl of the sample was used for DNA purification in a MagnaPure instrument. DNA was eluted in 100µl elution buffer and stored at -20°C until PCR amplification.

The V1-V2 hypervariable region were specifically targeted for PCR amplification using the primers sets (27F-AGAGTTTGATCCTGGCTCAG; R357-CTGCTGCCTYCCGTA). 2µl of DNA was used in a 25µ PCR reaction volume in triplicates containing 1 x fast start high fidelity buffer (Roche, Mannheim, Germany), 1.25 U high fidelity enzyme (Roche, Mannheim, Germany), 200 µM dNTPs (Roche, Mannheim, Germany), 0.4 µM barcoded primers and PCR-grade water (Roche, Mannheim, Germany). DNA was amplified in cycling conditions of 95°C for 3 minutes, 30 cycles: 95°C for 45 seconds, 55°C for 45 72°C for 1 minute, final extension at 72°C for 7 minutes. Triplicates were pooled, checked on a 1%

agarose gel, and 15µl of the pooled PCR product was normalized according to the manufacturer's instructions on a SequalPrep Normalization Plate (Life Technologies). 15µl of the normalized PCR product was used as the template for indexing PCR in a 50µl single reaction (composition as described for the targeted PCR) to introduce barcode sequences to each sample. Cycling conditions were the same as for the targeted PCR with only 8 cycles for amplification. After indexing 5µl of each sample were pooled, 50µ of the un-purified library were loaded to a 1% agarose gel and purified from the gel with a Qiaquick gel extraction kit (Qiagen, Hilden, Germany) according to manufacturer's instructions.

The pool was quantified using the QuantiFluor ONE dsDNA Dye on the Promega Quantus instrument according to the manufacturer's instructions and the size of the sequencing library was validated on an Agilent 2100 Bioanalyzer (Agilent) using a high sensitivity DNA assay according to manufacturer's instructions. The pool containing all samples was run at 6 pM final concentration with version 3 600 cycles chemistry (Illumina, Eindhoven, Netherlands) according to manufacturer and with 20% PhiX control DNA (Illumina, Eindhoven, The Netherlands). To obtain a unique combination of bar-code sequences, a second amplification step was performed using the Illumina nextera forward and reverse adaptor primers (Illumina, San Diego, CA, USA). Samples were sequenced using an Illumina MiSeqTM platform, following the manufacturer's specifications, generating 300 base-length paired-end reads.

Metabolomics

For metabolite extraction, thirty to fifty mg of each stool sample were thawed and 400 µL ice-cold methanol and 200 µL MilliQ H_2O were added to each sample. Stool samples were transferred to 2 mL tubes with O-ring caps containing Precellys beads (1.4 mm zirconium oxide beads, Bertin Technologies, Villeurbanne, France) for homogenization by Precellys24 tissue homogenizer (Bertin Technologies, Mon-tigny-le-Bretonneux, France). After centrifugation at 13,000 rpm for 45 min (4 °C), the supernatant was transferred to new 1.5 mL tubes and subsequently lyophilized at <1 Torr, 850 rpm, 25 °C for 10 h in a vacuum-drying chamber (Savant Speedvac SPD210 vacuum concentrator) with an attached cooling trap (Savant RVT450 refrigerated vapor trap) and vacuum pump (VLP120) (all Thermo Scientific, Waltham, MA). For plasma and urine metabolite extraction, 400 µL ice-cold methanol were added to 200 µL plasma or urine, respectively and stored at -20°C for 1 h. Afterwards the samples were spun at 17,949 rcf at 4°C for 30 min. Supernatants were lyophilized as described above for stool samples. For the NMR experiments, the samples were re-dissolved in 500 µL of NMR buffer (0.08 M Na_2HPO_4, 5 mM TSP (3-(trimethylsilyl) propionic acid-2,2,3,3-d_4 sodium salt), 0.04 (w/v)% NaN_3 in D_2O, pH adjusted to 7.4 with 8 M HCl and 5 M NaOH) and transferred to 5 mm NMR tubes for data acquisition.

Metabolic-profiling analysis was conducted at 310 K using a 600 MHz Bruker Avance Neo NMR spectrometer (Bruker Biospin, Rheinstetten, Germany) equipped with a TXI 600S3 probe head. The

Carr–Purcell–Meiboom–Gill (CPMG) pulse sequence was used to acquire ^{1}H 1D NMR spectra with a pre-saturation for water sup-pression (cpmgpr1d, 512 scans, 73,728 points in F1, 12019.230 Hz spectral width, 1024 transients, recycle delay 4 s). NMR spectral data were processed as previously described.(1) Briefly, data were processed in Bruker Topspin version 4.0.2 using one-dimensional exponential window multiplication of the FID, Fourier transformation, and phase correction. The NMR data were then imported into Matlab2014b; TSP was used as the internal standard for chemical-shift referencing (set to 0 ppm); regions around the water, TSP, and methanol signals were excluded. The NMR spectra were aligned, and a probabilistic quotient normalization was performed. Reduced spectra and normalized spectra were generated by Matlab2014b, among which the normalized spectra were used to quantify metabolites by signal integration. For each quantified metabolite, a characteristic peak without interfering signals was selected, the start and end points limiting the range of the peak were defined to integrate the area of the peak by summing the values for each point.

The integrations were used for PCA, orthogonal partial least squares discriminant analysis (O-PLS-DA) including associated data consistency checks and cross-validation, using MetaboAnalyst 5.0. (2) The statistical significance of the identified differences was validated by the quality assessment statistic Q^2.

Neutrophil function

Neutrophil function was assessed by flow cytometry in heparinized whole blood using Phagoburst kits (Celonic, Basel, Switzerland) according to manufacturer's instructions. In short: 100µl of heparinized whole blood was incubated with either PBS, FMLP or E.coli for 10 minutes at 37°C. The reactive oxygen species produced in this time oxidise the added dihydrorhodamine 123 to rhodamine 123 which emits a green fluorescent signal. Flowcytometry was used to measure the amount of fluorescent signal in approximately 10.000 neutrophils. The percentage of positive cells and the geometric mean of fluorescence intensity in the positive cells were used to quantify resting burst (ROS production without additional stimuli/PBS), priming (ROS production after stimulation with fMLP) and oxidative burst (ROS production after stimulation with heat inactivated E. coli bacteria)

Supplementary Results

To assess the potential confounding effect of lactulose, PPI and rifaximin we performed a subgroup analysis of the 16s rDNA sequencing data without these patient. This analysis showed that Flavonifractor and Oscillospira are still higher in the LOLA group than in the control groups. For details see the following figure (A-B). Similar subgroup analysis was done for PPI (C-D) and rifaximin (E-F) with similar results. Due to the low power and robustness these results have to be viewed with caution.

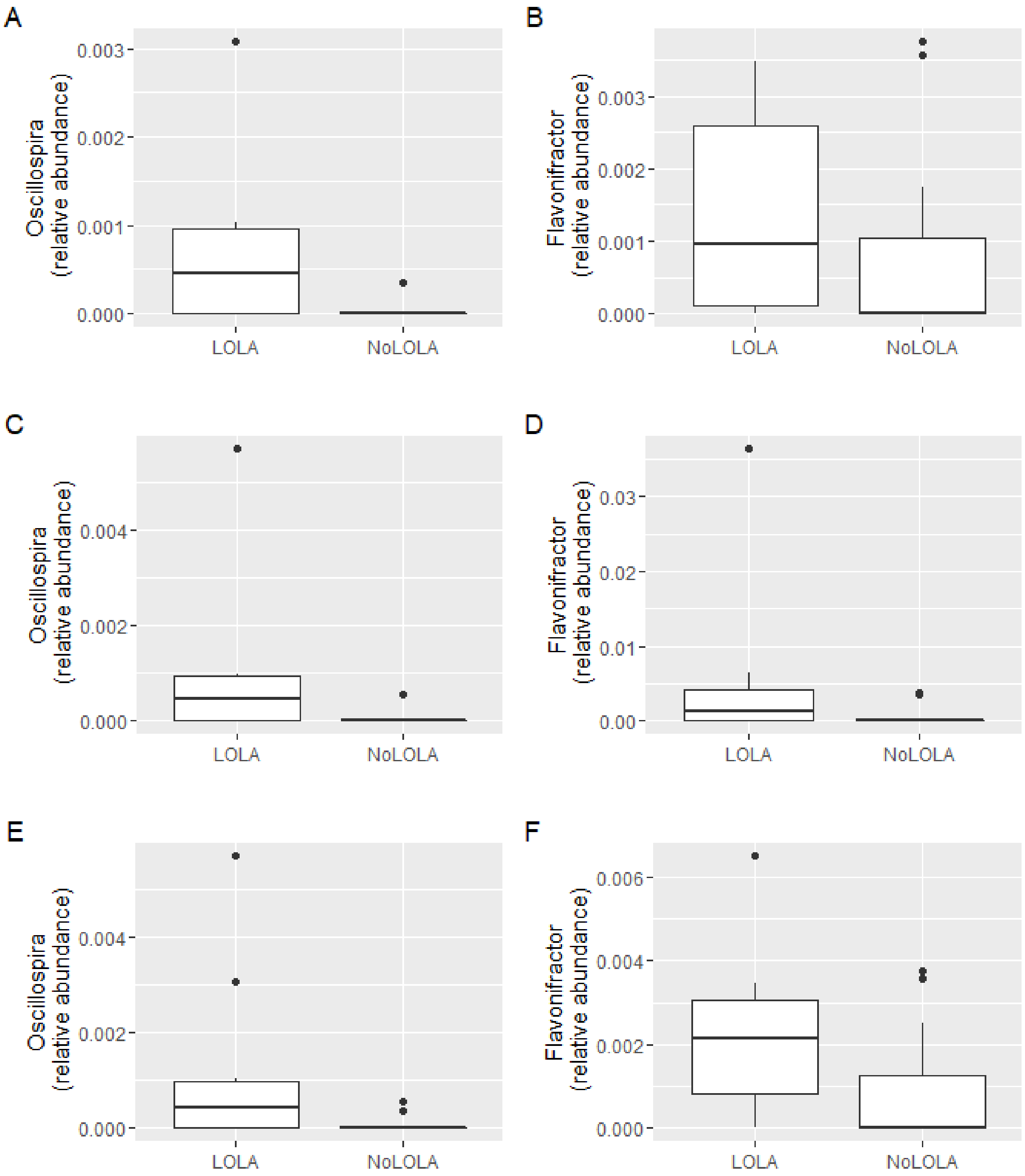

Figure S1. Relative abundance of Oscillospira and Flavonifractor in patients with and without LOLA use and no concomitant lactulose use (A-B), no concomitant PPI use (C-D) and no concomitant rifaximin use (E-F).

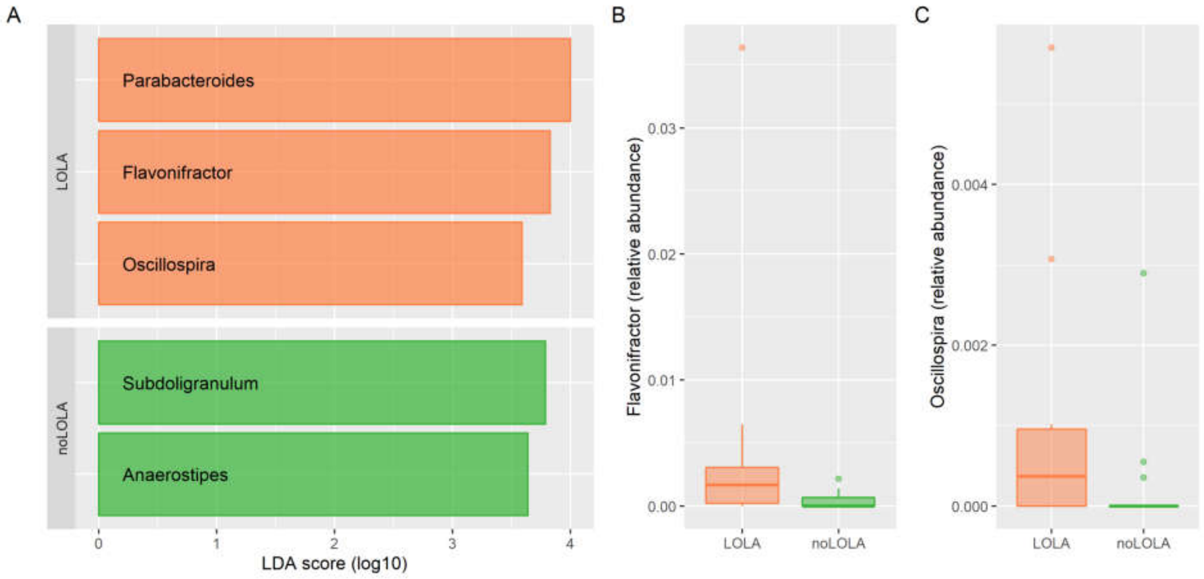

Figure S2. Sensitivity analysis A) Genera associated with LOLA therapy (orange bars - LOLA) or controls (green bars - noLOLA) determined by LDA Effect Size (LEfSe) in the sensitivity analysis. B-C) Relative abundances of genera *Flavonifractor* and *Oscillospira* in patient with and without LOLA intake.

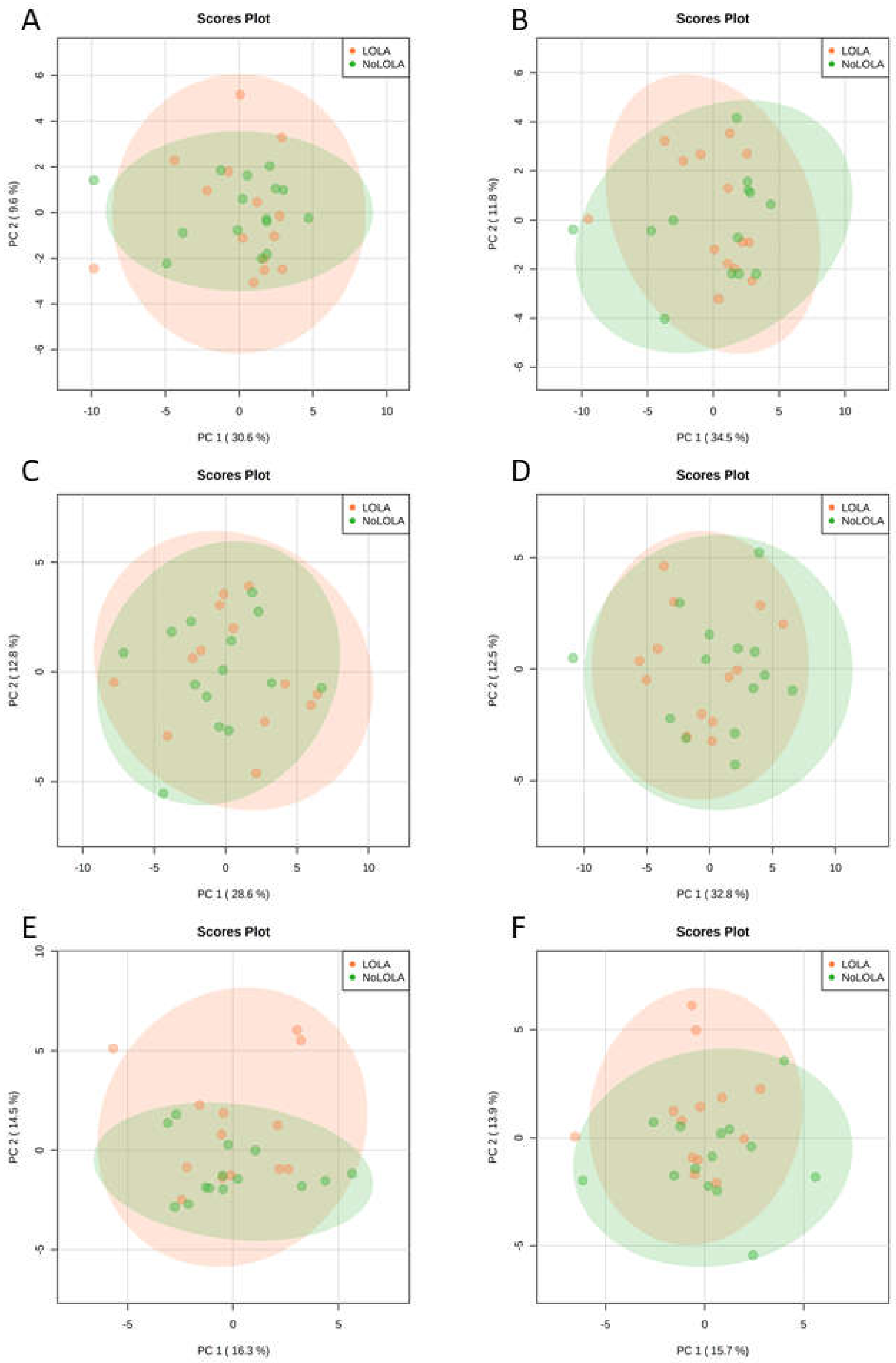

Figure S3. A+B) Similarity of urine metabolome in patients with and without LOLA. Scores plot of principal component analysis based on the urine metabolome in the initial analysis (A) and the

sensitivity analysis (B). C+D) Similarity of stool metabolome in patients with and without LOLA. Scores plot of principal component analysis based on the stool metabolome in the initial analysis (C) and the sensitivity analysis (D). E+F) Similarity of serum metabolome in patients with and without LOLA. Scores plot of principal component analysis based on the serum metabolome in the initial analysis (E) and the sensitivity analysis (F).

Table S1. Targeted metabolomics including liver disease parameters, gut inflammation and permeability markers, indicators of sarcopenia and neutrophil function. Values are given in mean (standard deviation). Significant differences are marked in bold print.

**Parameter**	**LOLA (n=15)**	**noLOLA (n=15)**	**p-value**
Alanine aminotransferase (U/L)	38.8 (22.78)	43 (32.97)	0.9
Aspartate aminotransferase (U/L)	67.67 (41.89)	62.47 (35.92)	0.8
Alkaline phosphatase (U/L)	137.67 (59.17)	127.67 (50.76)	0.7
Gamma-glutamyltransferase (U/L)	134.47 (92.78)	163.73 (118.2)	0.5
Albumin (g/dL)	3.19 (0.51)	3.47 (0.61)	0.1
Bilirubin (mg/dL)	2.26 (1.8)	2.04 (2.18)	0.2
Prothrombin time internaional normalized ratio	1.38 (0.23)	1.26 (0.29)	0.2
Total protein (g/dL)	6.83 (0.95)	7.27 (0.81)	0.08
Fecal calprotectin (ng/mL)	101.37 (103.88)	81.41 (54.69)	0.9
Fecal zonulin (ng/mL)	161.2 (219.87)	99.61 (42.24)	0.4
Diamine oxidase (U/mL)	23.99 (11.94)	20.45 (14.97)	0.3
LPS binding protein (µg/mL)	16.74 (6.99)	23.56 (15.8)	0.3
C-reactive protein (mg/L)	10.32 (13.56)	10.55 (17.82)	0.4
**soluble Cluster of Differentiation 14 (µg/mL)**	1.78 (0.43)	2.24 (0.94)	0.1

Parameter	LOLA (n=15)	noLOLA (n=15)	p-value
Fibroblast growth factor 21 (ng/ml)	0.35 (0.64)	0.75 (1.23)	0.08
Irisin (µg/mL)	2.01 (1.46)	2.32 (1.03)	0.2
Myostatin (ng/mL)	44.03 (34.1)	42.03 (11.98)	0.3
Insulin-like growth factor 1 (ng/mL)	48.3 (28.06)	72.91 (41.57)	0.06
Chair rise test (s)	24.98 (15.89)	20.31 (8.11)	0.9
Gait speed (m/s)	0.84 (0.3)	0.99 (0.26)	0.2
Midarm muscle circumference (mm)	256.58 (63.7)	246.54 (39.75)	0.7
Hand grip strength (kg)	30.64 (10.56)	28.26 (8.88)	0.5
Body mass index (kg/m^2)	27.48 (6)	26.15 (5.55)	0.4
Resting burst of neutrophils (% of neutrohils)	2.39 (0.93)	2.15 (0.98)	0.4
Resting burst of neutrophils (GMFI)	173.97 (102.91)	188.99 (137.01)	0.5
Neutrophil priming (% of neutrophils)	3.15 (1.38)	2.6 (1.13)	0.3
Neutrophil priming (GMFI)	148.71 (58.67)	142.66 (37.56)	0.9
ROS production after E.coli stimulation (% of neutrophils)	96.73 (5.18)	98.31 (2.23)	0.5
ROS production after E.coli stimulation (GMFI)	1034.24 (547.1)	774.13 (320.74)	0.2

LOLA: patients with LOLA intake; noLOLA: patients without LOLA intake; LPS: lipopolysaccharide; GMFI: geometric mean of fluorescence intensity

Supplementary References

1. Stryeck S, Gastrager M, Degoricija V, Trbusic M, Potocnjak I, Radulovic B, et al. Serum Concentrations of Citrate, Tyrosine, 2- and 3- Hydroxybutyrate are Associated with Increased 3-Month Mortality in Acute Heart Failure Patients. Sci Rep. 2019;9(1):6743.

2. Pang Z, Chong J, Zhou G, de Lima Morais DA, Chang L, Barrette M, et al. MetaboAnalyst 5.0: narrowing the gap between raw spectra and functional insights. Nucleic Acids Res. 2021;49(W1):W388-W96.
